# Enhanced chromatin compaction is associated with de novo expression of a nuclear microprotein, global loss of H3 acetylation and local transcriptional changes in retinal rod photoreceptors

**DOI:** 10.21203/rs.3.rs-10017071/v1

**Published:** 2026-08-27

**Authors:** Claire Marchal, Mohita Gaur, Anjani Kumari, Sonali V. Mohan, Matthew J. Brooks, Catherine Jaeger, Jayshree Advani, Laura Campello, Milton A. English, Nivedita Singh, Ximena Corso-Díaz, Anand Swaroop

**Affiliations:** 1 Neurobiology, Neurodegeneration & Repair Laboratory, 6 Center Drive, MSC0610, National Eye Institute, National Institutes of Health, Bethesda, MD 20892, USA; 2 In silichrom ltd, 15 Digby Road, RG14 1TS, Newbury, UK

## Abstract

We have limited understanding of how aging alters gene expression and remodels cellular architecture in post-mitotic neurons. The inverted nuclear organization of mouse rod photoreceptors provides a unique model to gain mechanistic insights into age-associated decline in neuronal function. We have generated and integrated multi-omic datasets including 3D-genome topology, histone modifications, chromatin accessibility, DNA methylation and transcriptome of rod photoreceptors from young- and aged-mice. We show that aging drives global chromatin compaction, with regional alterations enriched at active chromatin. Epigenomic and transcriptional changes broadly correlate with chromatin dynamics as validated by high resolution microscopy. We uncover a megabase-sized genomic region with multi-level alterations, including *de novo* transcription of Gm7239, which encodes a functional microprotein carrying histone acetyltransferase-inhibitor domain. Overexpression of Gm7239 is associated with global loss of histone H3 acetylation, highlighting a potential new axis of genomic regulation in aging. Finally, we identify multiple significant local transcriptional alterations in non-annotated regions and genes associated with age-related macular degeneration. Our studies link age-related chromatin landscape changes with gene expression that may influence rod function and vulnerability to diseases.

## Introduction

The universality and intrigue of aging, despite tremendous variability in organismal lifespan, have led to evolutionary arguments and conceptual discussions over centuries. Nonetheless its definition remains debatable, and underlying causes remain poorly understood ^1^. In humans, advanced age profoundly impacts health and greatly enhances the risk for chronic diseases, affecting diverse tissues and physiological functions. Similar to age-associated pathologies, biological aging exhibits highly variable molecular and cellular phenotypes (hallmarks) and is determined by genetic factors as well as epigenomic alterations ^2–5^. Some of these changes likely develop from increased entropy over time, inherent to any dynamic system, whereas others reflect cellular adaptations that help preserve biological function. Large-scale genetic studies have begun to unravel variants that are associated with lifespan and age-related phenotypes ^3,6,7^; however, a direct contribution of aging to specific pathologies has been difficult to dissect as of yet. At the genomic level, aging leads to genomic instability, epigenome alterations (such as DNA methylation) and aberrant gene expression patterns including an increase in transcriptional noise ^2,4,8,9^. Epigenetic alterations, such as cytosine methylation and histone modifications, are inherently linked to metabolic networks and metabolites. Aging is also accompanied by decrease in mitochondrial function and NAD+ levels and disrupted protein homeostasis, which can further contribute to disease phenotypes ^4,10–13^. Genomic, epigenomic and metabolic hallmarks likely represent both causes as well as consequences of aging and form intricate networks of molecular aberrations and adaptations that progressively intensify with advancing age.

In mammals, long-lived post-mitotic neurons are particularly susceptible to stochastic and adaptive changes in cellular processes as well as to accumulation of macromolecular damage with age. Within the central nervous system, the retina is a highly metabolically active tissue that can quickly adapt to its continuously changing environment ^14–16^. Disruption in metabolic and epigenetic landscape in the retina contributes to pathological manifestations, including during aging, and metabolic and/or epigenetic reprogramming may result in restoration of retinal function ^10,17–20^. In concordance, integration of retinal transcriptome and methylome with genome-wide association studies has uncovered gene-epigenome interactions at genes associated with metabolic pathways, where the effect of age-related macular degeneration (AMD)-linked genetic variants is likely mediated via changes in DNA methylation and gene expression ^21^.

The rod and cone photoreceptors in the mammalian retina have high energy requirements, and of these, rods are especially vulnerable to aging and disease conditions ^22–25^. Rods in nocturnal mammals, such as mice, exhibit a unique nuclear architecture with heterochromatin concentrated in the center and euchromatin at the periphery, directly affecting the entry of light by acting as light collection lenses ^26,27^. The spatial segregation of heterochromatin and euchromatin also makes mouse rods an ideal model for visualizing and quantifying age-related changes across chromatin landscape through microscopy. Using an engineered mouse line expressing GFP specifically in rods ^28^, we had demonstrated global changes in DNA methylation in aging rods and provided evidence of disruption in metabolic homeostasis ^29^. However, we have poor understanding of chromatin architecture and epigenome in the aging retina, including rod photoreceptors, and how molecular changes translate to visual dysfunction in aging and associated phenotypes.

Cell fate and gene expression in the mammalian rod photoreceptors are largely determined by Maf-family basic motif leucine zipper (bZIP) protein NRL (Neural Retina Leucine Zipper) in concert with multiple regulatory proteins ^30–32^. We and others have generated transcriptome profiles and mapped epigenetic landscape as well as genome topology of developing and mature human and mouse retina ^33–39^. Here we report qualitative as well as quantitative analyses of age-related chromatin alterations by combining multiple layers of omics data obtained from a pure population of post-mitotic mouse rod photoreceptors. Taking advantage of the inverted chromatin of the mouse rods, we validate our findings using microscopy, ELISA, immunoblotting and targeted mass-spectrometry experiments. We have uncovered increased chromatin compaction during aging, concomitantly to a decrease in pan-histone H3 acetylation. Local age-related chromatin changes occur predominantly at active chromatin, including a large genomic region with *de novo* transcription of Gm7239, which encodes a functional histone acetyltransferase (HAT) inhibitor. We discover major age-related local changes in transcription at non-annotated regions and at genes associated with AMD. Our studies show that aging alters chromatin dynamics to produce specific changes in gene regulation.

## Results

### Multi-omics data integration captures age-associated chromatin landscape

**Figure 1:**
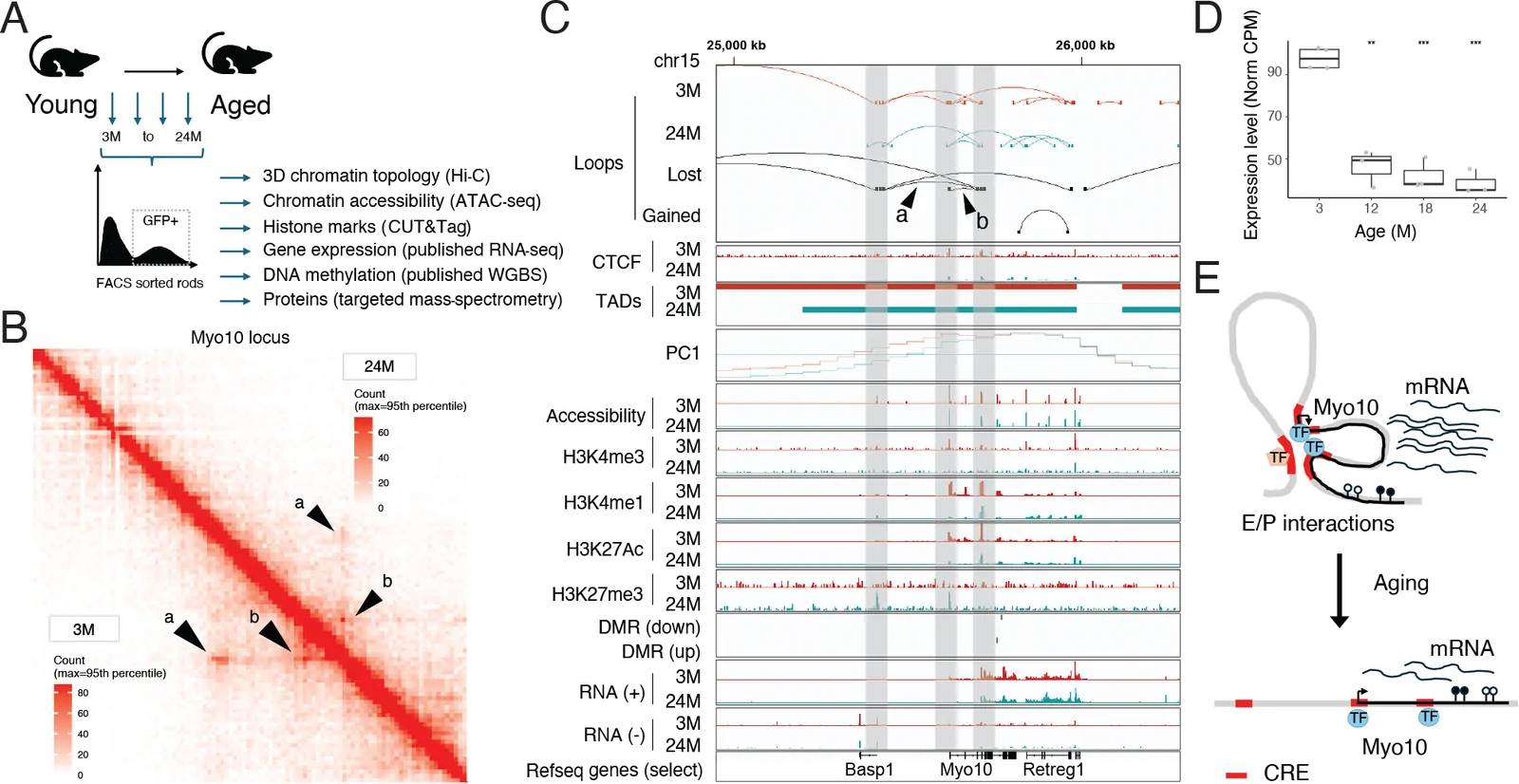
Multi-omics chromatin profiling of mouse rods reveals changes in chromatin landscape during aging. **A.** Schematics of the experimental design. **B.** Contact map at the Myo10 locus for 3- (bottom triangle) and 24-month (top triangle) mouse rods. Arrows **a** and **b** point to two chromatin loops present at 3- and absent at 24-month. **C.** Chromatin profile at a region centered on the Myo10 gene. Tracks represent from top to bottom: chromatin loops, CTCF coverage, TADs, compartments (PC1), chromatin accessibility (log scale), H3K4me3, H3K4me1, H3K27ac and H3K27me3 coverages, differentially methylated regions (DMR) and transcription on + strand and − strand at 3- (red) or 24-months old (blue), and a selection of RefSeq genes. Arrows **a** and **b** point to the two chromatin loops highlighted in **B.** Vertical grey rectangles point to the regulatory regions at the feet of loops **a** and **b**. Genomic coordinates in mm10 are reported here. **D.** Expression level from RNA-seq data (NormCPM) of Myo10 gene at different ages (n = 3 to 4). Grey dots represent the value for each individual replicate. Boxplots represent the median and interquartile range (IQR); whiskers mark 1.5x the IQR; data beyond 1.5x the IQR are plotted as individual points. Statistics by t test: ** is p-value < 0.01, *** is p-value < 0.001. **E.** Schematics of Myo10 expression regulation in aging.

To measure the extent of age-related changes in 3D chromatin organization, we first performed high resolution Hi-C on freshly isolated rod photoreceptors from 5 biological replicates of retinas of young mice (2- to 4-month, hereafter referred as 3-month) and aged mice (over 22-month, hereafter referred as 24-month) mice (Supplementary Figure 1A and Supplementary Table 1). The Hi-C data reveals strong correlation between biological replicates and displays interaction patterns comparable to previously published adult rod profiles ^34^, but with higher resolution (merged replicates in our study reach a 2.4 kb (3-month) and 2.8 kb (24-month) resolution on chr1, Supplementary Figures 1B-F). The genome topology data was integrated with newly generated ATAC-seq and CUT&Tag data from 3- and 24-month mouse rods (Figure 1A) as well as with our previously published RNA-seq and Whole Genome Bisulfite Sequencing (WGBS) ^29^. We detect clustering of replicates by age (Supplementary Figures 1G and H) when measuring chromatin accessibility and genomic occupancy of CTCF and several histone marks (H3K27ac, H3K27me3, H3K4me1 and H3K4me3). At local levels, we observe age-related changes of chromatin landscapes. An example of such changes is the *Myo10* gene, showing robust chromatin landscape alterations captured in our dataset (Figures 1B and C). At this locus, two chromatin loops between the *Myo10* gene promoter and two other regulatory regions with multiple active histone marks are almost completely lost in 24-month rods (Figures 1B and C; see arrows a and b). We also identify concomitant loss of accessibility as well as histone marks for active/paused chromatin states (H3K4me1, H3K27ac and H3K4me3) and gain of repressive marks (H3K27me3) at the regulatory regions that overlap with the lost loop feet (Figure 1C, grey rectangles). In parallel, *Myo10* expression decreases significantly with age (Figure 1D). Integration of multi-omics data highlights co-occurring age-related changes in chromatin landscape and gene expression (Figure 1E) at multiple specific loci, suggesting a key role of chromatin remodeling in functional decline of rod cells during retinal aging.

### Rod chromatin exhibits a global compaction with aging

**Figure 2:**
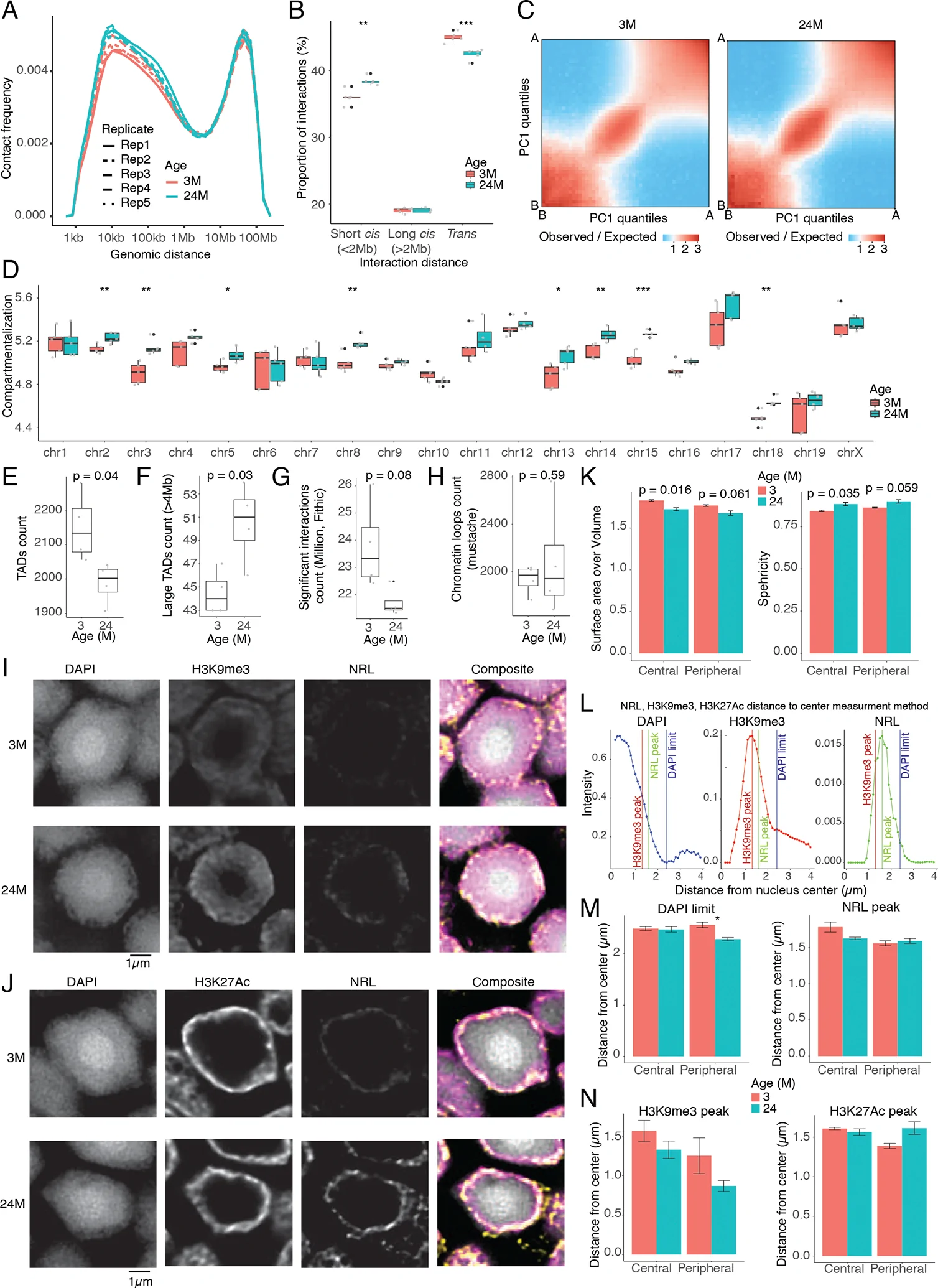
Aging leads to a global compaction of rod chromatin. **A.** Distribution of the interactions distance. Data are plotted from 10 M randomly picked interactions per sample (n = 5). **B.** Quantification of short, long range *cis*-interaction and *trans*-interactions using 10M randomly picked interactions (n = 5). **C.** Saddle plot of intra- and inter-compartment interactions during aging in merged replicates. Color scale shows the ratio of observed over expected interaction count between compartment quantiles. **D.** Compartmentalization per chromosome during aging, calculated as the ratio of intra- over inter-compartment interactions (n = 5). **E.** TAD count at 3 vs 24 month (n = 4). **F.** Large TADs (>4Mb) count at 3 vs 24 month (n = 4). **G.** Significant interactions (assessed using Fithic) at 3 vs 24 months (n = 4). **H.** Loop count at 3 vs 24 months assessed with mustache (n = 4). **I-J.** Confocal imaging of rod nuclei at 3 and 24 months showing DAPI, H3K9me3 (**I.**) or H3K27ac (**J.**) and NRL signal. **K.** Surface area over volume ratios (left) and sphericity (right) of filtered rods nuclei measured by confocal imaging. Nuclei are filtered for center within 1μm from x, y and z image borders and volume within 20 to 40 μm. X axis represents the part of the retina. Values represent the mean of the filtered nuclei and error bars represent the standard error of the means across the biological replicates (n = 3). **L.** Method of computation of the distance from the nucleus center of NRL, H3K9me3 and H3K27ac maximum intensity peak: example of one nucleus is shown. **M.** Mean of DAPI maximum distance (left) or NRL intensity peak distance (right) from centers of filtered nuclei. X axis represents the part of the retina. Values represent the mean of the filtered nuclei and error bars represent the standard error of the means across the biological replicates (n = 3). **N.** Mean of H3K9me3 (left) or H3K27ac (right) intensity peak distance from centers of filtered nuclei. X axis represents the part of the retina. Values represent the mean of the filtered nuclei and error bars represent the standard error of the means across the biological replicates (n = 3). Boxplots represent the median and interquartile range (IQR); whiskers mark 1.5x the IQR; data beyond 1.5x the IQR are plotted as individual points. Statistics by t test: * is p-value<0.05, ** is p-value < 0.01, *** is p-value < 0.001. Grey dots represent the value for each individual replicate.

Given that chromatin contact maps uncover both large-scale and local changes (Supplementary Figure 2A and B), we examined age-associated chromatin architecture by quantifying genome-wide distribution of 3D chromatin interaction distance. We noted significant changes in 24-month rods compared to 3-month rods, with increase in short-range interactions (< 2Mb) and decrease of trans-interactions (Figures 2A and B). We also detect an increase in compartmentalization strength with age for most of the chromosomes (Figure 2C with quantification in Figure 2D; Supplementary Figure 2C), reflecting enhanced segregation of chromatin compartments that can potentially influence gene expression patterns.

A global increase in chromatin compartmentalization, associated with an increase in short-range interactions, indicates a more compact chromatin in aging rod nuclei. Accordingly, we detect a significant decrease in the number of TADs and an increase in large TADs (> 4 Mb) (Figures 2E and F, Supplementary Figure 2D), suggesting the merger of some TADs by 24-month. At the finest level, the number of significant interactions (i.e., contacts at significantly higher level than expected in one given sample) tend to decrease though not significantly, whereas chromatin loop counts remain unchanged in 24-month rods (Figures 2G and H, Supplementary Figure 2E).

We then analyzed photoreceptor nuclei from 3- and 24-month mice by confocal microscopy covering a total of 5 μm z-stacks (Figures 2I and J; Supplementary Figures 3A and B). After filtering nuclei for position and volume, 24-month rod nuclei are significantly more compact (lower surface area to volume ratio) and tend to be more spherical compared to 3-month nuclei (Figure 2K). We took advantage of the unique inverted chromatin structure in the mouse rod nuclei and measured the average distance of heterochromatin and euchromatin relative to the nucleus center based on different markers. We computed the median signal intensity for DAPI as well as NRL, H3K9me3 and H3K27ac around each nucleus center. The positions of NRL, H3K9me3 and H3K27ac peak intensity were based on DAPI-defined boundary. Figure 2L shows an example of the signal for one nucleus (see also Supplementary Figures 4A-B). Though these distances do not significantly change in aging, the peak of H3K9me3 signal tends to be closer to the nucleus center in 24- month rods compared to the 3-month (Figures 2M–N).

Altogether our data demonstrate that mouse rod chromatin undergoes a global compaction at advanced age, consistent with previous observations in senescent fibroblasts ^40^.

### Age-associated local chromatin reorganization occurs predominantly in active chromatin

**Figure 3:**
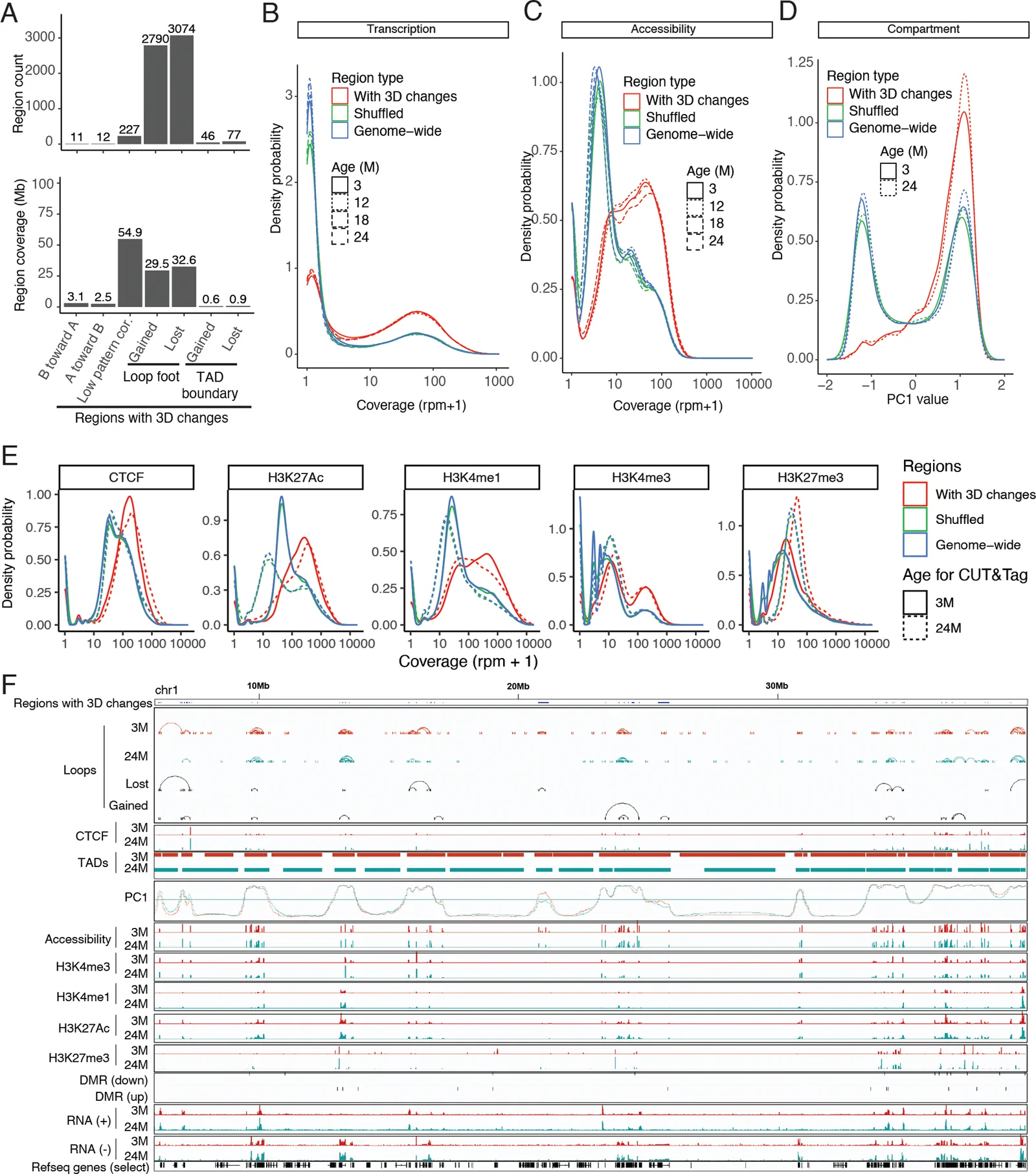
Age-associated local chromatin topology changes occur predominantly at active chromatin. **A.** Characterization of regions with change in 3D chromatin topology: region count (top) and total genome coverage (Mb, bottom) **B-D.** Transcription coverage (read per million, rpm, +1, **B.**), accessibility (read per million, rpm, +1, **C.**) and compartment score (PC1, **D.**), distribution in 50kb windows overlapping regions with 3D changes identified in **A.** (red), shuffled regions (green) or genome-wide (blue) in 3- and 24-month. **E** CTCF, H3K27ac, H3K4me1, H3K4me3 and H3K27me3 coverages (read per million, rpm, +1) distribution in 50kb windows overlapping regions with 3D changes identified in **A.** (red), shuffled regions (green) or genome-wide (blue) in young and old mice. **F.** Chromatin profile on an example region. Tracks represent from top to bottom: regions with 3D changes identified in **A.**, chromatin loops, CTCF coverage, TADs, compartments (PC1), chromatin accessibility (log scale), H3K4me3, H3K4me1, H3K27ac and H3K27me3 coverages, differentially methylated regions (DMR) and transcription on + strand, transcription on − strand at 3- (red) or 24-months old (blue), selection of RefSeq genes. Genomic coordinates in mm10.

We next assessed whether local changes in 3D chromatin conformation occur genome-wide or are restricted to specific regions. We identified several types of 3D chromatin alterations in 24-month rod nuclei: 12 regions strongly switched from A to B compartments, and 11 from B to A; 46 TAD boundaries were acquired and 77 lost; 2,790 loops were gained and 3,074 lost; and 227 regions showed changes in local interaction patterns (Figure 3A; Supplementary Figures 5A-G, see methods for more information about local interaction patterns). Most of these regions do not directly overlap, except for changes in compartments that are a perfect subset of the regions with change of global pattern of interactions (Supplementary Figure 5H). These results highlight the complementarity of different approaches to identify 3D reorganization. Altogether, these regions represent over 100 Mb of the genome.

Chromatin landscape at the altered local interaction regions shows strong enrichment for higher transcription, accessibility, and the A compartment compared to the random shuffled regions or genome-wide (Figures 3B–D, Supplementary Figures 6A-C). Moreover, these regions are enriched for CTCF binding sites and active histone marks (Figure 3E, Supplementary Figure 6D). This enrichment at active chromatin is evident when visualizing large genomic regions such as a segment of chromosome 1 (Figure 3F).

We conclude that age-related 3D chromatin interaction changes occur predominantly at active chromatin.

### Local changes in topology overlap altered chromatin landscape and transcription

**Figure 4:**
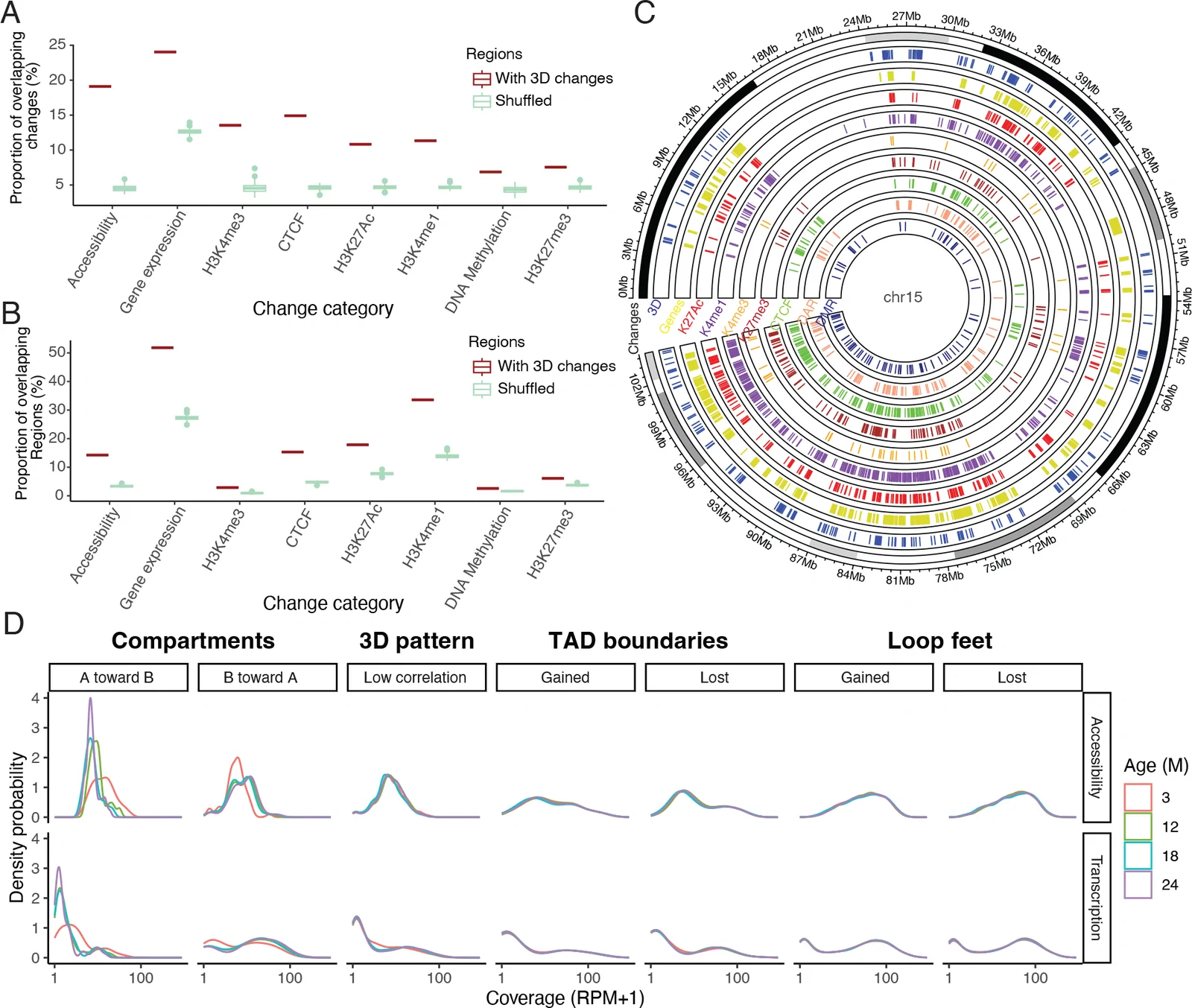
Age-associated changes in 3D organization overlap with changes in transcription, chromatin accessibility, DNA methylation and histone marks. **A.** Proportion differential chromatin landscape loci (differential accessibility, gene expression, histone marks, CTCF binding and DNA methylation) overlapping regions with 3D changes (dark red) or 100 sets of shuffled regions (light green). **B.** Proportion of regions with 3D changes (dark red) or 100 sets of shuffled regions (light green) overlapping with differential chromatin landscape loci (differential accessibility, gene expression, histone marks, CTCF binding and DNA methylation). **C.** Circos plot of chromosome 15. Tracks represent (toward center of the circle) 3D changing regions (“3D”), DE genes (“Genes”), Differential H3K27ac (“K27Ac”), H3K4me1 (“K4me1”), H3K4me3 (“K4me3”), H3K27me3 (“K27me3”) and CTCF (“CTCF”), accessible (“DARs”) and methylated (“DMRs”) regions. Genomic coordinates in mm10. **D.** Accessibility and transcription coverages (read per million, rpm, +1) at different subsets of regions with 3D changes at 3- and 24-months. Boxplots represent the median and interquartile range (IQR); whiskers mark 1.5x the IQR; data beyond 1.5x the IQR are plotted as individual points.

To further evaluate chromatin features, we computed differences in chromatin accessibility (Supplementary Figure 7A), histone marks and CTCF binding (Supplementary Figure 7B). Even though overall changes are specific for each mark, genomic regions having multiple alterations tend to have gain in active marks associated with loss of repressive marks or loss in active marks associated with gain in repressive marks (Supplementary Figure 7C). All regions with 3D chromatin changes in aging rods are enriched for differentially expressed genes (DE genes ^29^), differentially methylated regions (DMRs ^29^), differentially accessible regions (DARs) and differentially bound loci for H3K4me3, H3K27ac, H3K4me1 and CTCF compared to 100 sets of shuffled regions (Figure 4A). Notably, protein coding genes are enriched within these regions (Supplementary Figure 7D). Conversely, genomic regions exhibiting changes in other chromatin features are generally enriched for 3D chromatin alterations, with the exception of H3K4me3, H3K27me3, and DNA methylation (Figure 4B). Chromosome-wide, these changes cluster in the same genomic regions (Figure 4C). Interestingly, differences in global accessibility and transcription coverages are strongly associated with regions undergoing compartment changes, but not with other types of 3D changes (Figure 4D). This could, however, reflect the fact that regions switching from B to A compartments exhibit increased transcription and accessibility, whereas those switching from A to B compartments show the opposite trend. Unlike compartment shifts, the deviations in global pattern, TAD boundaries or loop feet are not necessarily linked to a directional change in transcription or accessibility. Transcription and accessibility appear to deviate between 3–12-month timepoints in mice and are complete by 12–24 month (see Figure 4D). Global coverage of histone marks also changes modestly at regions with compartments, with gain of H3K27ac and H3K4me1 at regions switching from B toward A (Supplementary Figure 7E). Thus, regions with changes in 3D chromatin landscape at least partially overlap with those in gene expression, highlighting redundancy in gene regulation.

### A megabase sized locus presents alterations in chromatin topology associated with position-dependent increase in transcription

**Figure 5:**
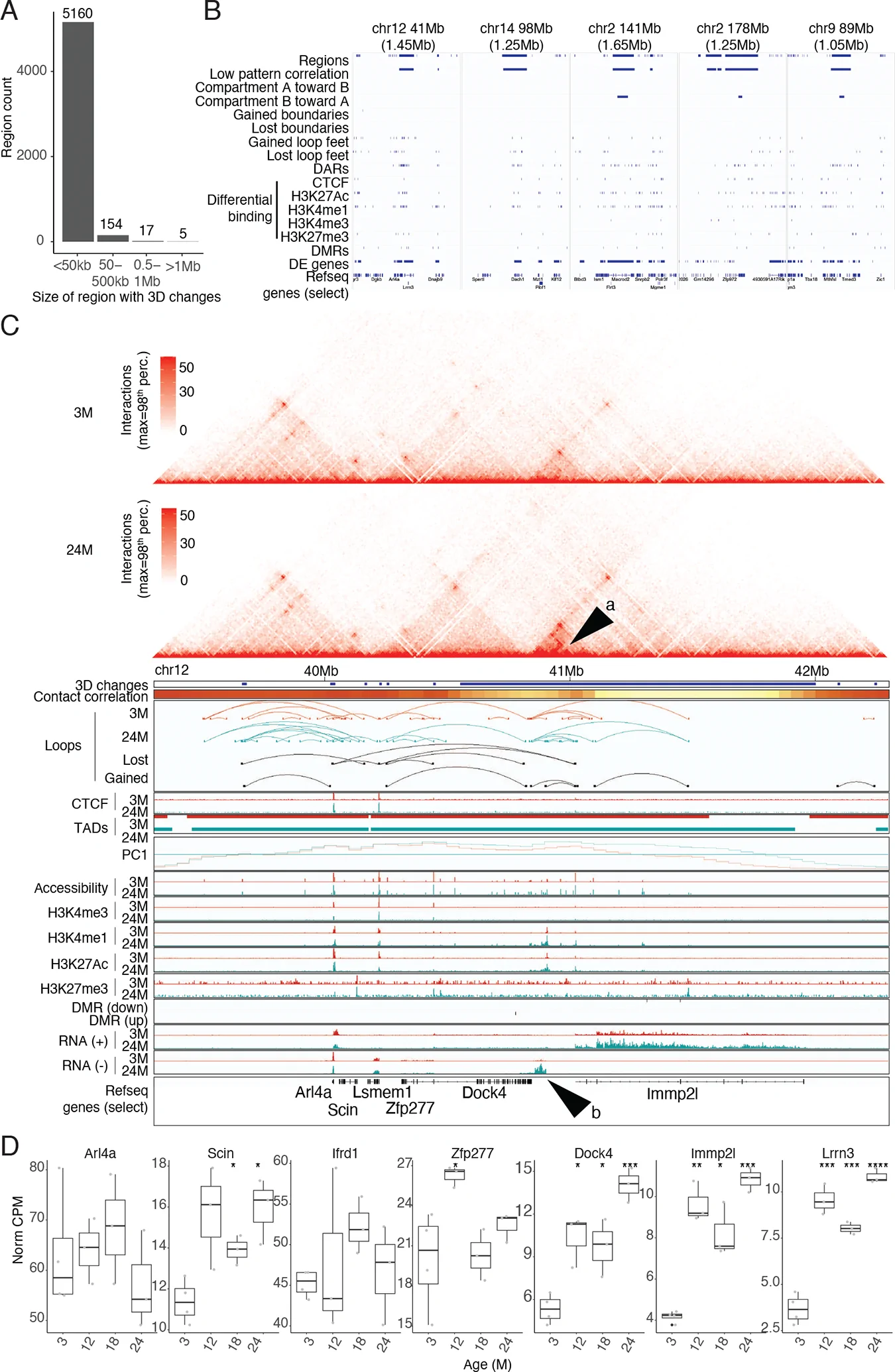
Dock4 region presents changes in chromatin landscape associated with position-dependent change in gene expression. **A.** Size distribution of regions with 3D changes. **B.** Changes in chromatin landscape at regions with 3D change over 1Mb length. Tracks represent from top to bottom: 3D changing regions, regions with low interaction pattern correlation, compartment changes, TAD boundary changes, loop feet changes, differentially accessible regions (DARs), differentially bound chromatin for CTCF, H3K27ac, H3K4me1, H3K4me3 and H3K27me3, differentially methylated regions (DMRs), DE genes and a selection of RefSeq genes. Genomic coordinates in mm10. **C.** Overview of the Dock4 locus on chr12. Tracks represent from top to bottom: chromatin contacts at 3 and 24 months, chromosome coordinates (mm10), regions with 3D changes, global pattern correlation between 3 and 24 months (yellow for low correlation, red for high correlation), chromatin loops, CTCF coverage, TADs, compartments (PC1), chromatin accessibility (log scale), H3K4me3, H3K4me1, H3K27ac and H3K27me3 coverages, differentially methylated regions (DMR) and transcription on + strand and − strand at 3- (red) or 24-months old (blue), and a selection of RefSeq genes. Black arrow **a** highlights a region showing strong increase of local chromatin contacts during aging, where black arrow **b** indicates a region showing strong increase of transcription during aging. **D.** Gene expression around the Dock4 locus. Genes are presented in the relative order they are in the locus. (n = 3 – 4) Boxplots represent the median and interquartile range (IQR); whiskers mark 1.5x the IQR; data beyond 1.5x the IQR are plotted as individual points. Statistics by t test: * is p-value<0.05, ** is p-value < 0.01, *** is p-value < 0.001. Grey dots represent the value for each individual replicate.

Majority of loci with altered 3D chromatin organization are under 50 kb, likely comprising of isolated loop changes. Remarkably, five regions with changes in 3D chromatin are over 1 Mb (Figure 5A). These regions are mostly defined by large domains of low correlation between 3- and 24-month interaction patterns, overlapping with other chromatin changes and with differentially expressed genes (Figure 5B). All these changes in chromatin landscape co-occur at the *Dock4* locus (Figure 5C). This locus also displays an interesting pattern of expression dynamics where genes from the distal but not the centromeric side of the extended locus present significantly higher expression between 3–12 months, except for the *Scin* gene (Figure 5D). These results point towards a local regulation for the whole group of genes on the distal side. Interestingly, a strong increase in chromatin contacts (Figure 5C, black arrow a) occurs with aging in a well-defined 50 kb area of the locus. Augmented contacts are associated with higher transcription in the same area, an intergenic locus downstream of *Dock4* gene (Figure 5C, black arrow b).

### Age-induced expression of a histone acetyl transferase inhibitor at the Dock4 locus correlates with global disruption of H3 acetylation

**Figure 6:**
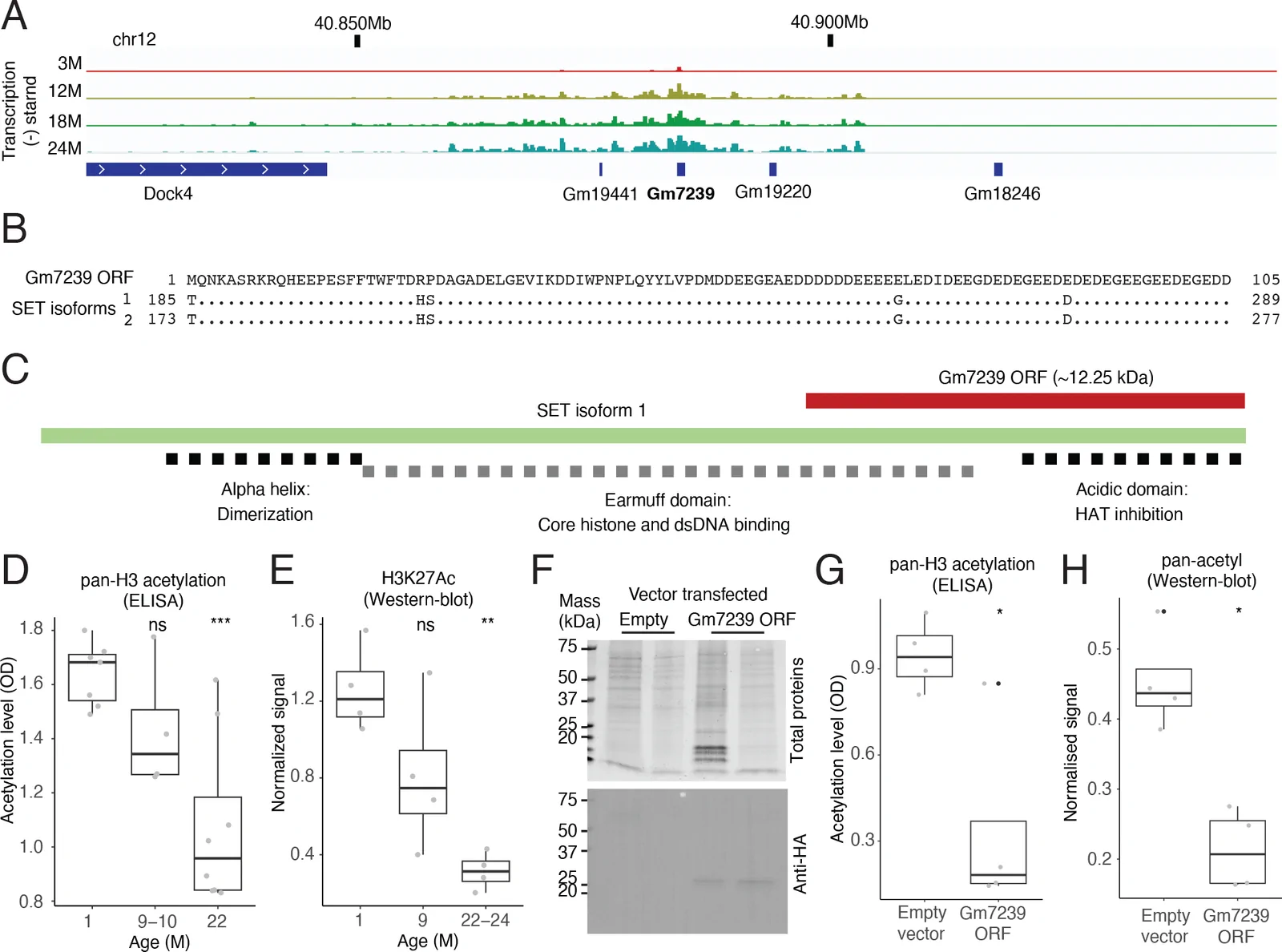
De-repression of a silenced HAT-inhibitor coding pseudo-gene during aging correlates with histone acetylation decrease. **A.** RNA coverage downstream of Dock4 coding gene. Normalized coverages on minus strand are shown for replicate #1 of 3-, 12-, 18- and 24-month mouse rods. **B.** Alignment between the predicted peptide sequence of the open reading frame (ORF) overlapping Gm7239 and the peptide sequences of SET isoforms 1 and 2. **C.** Schematics of SET protein isoform 1 protein domains (light green) and their overlap with the Gm7239 predicted ORF sequence (dark red). **D-E.** Measure of global histone H3 acetylation by ELISA (**D.**, n = 4 to 6) and H3K27ac level by Western-blot (**E.**, n = 4) during aging. Membranes are shown in supplementary figures. **F.** Exogenous expression of Gm7239 ORF coupled to HA tag in HEK293T cells transfected with empty vector or vector with Gm7239 ORF coupled to HA tag inserted (immunoblot of two replicates, all replicates are shown in supplementary figures). **G-H.** Quantification of total-H3 acetylation using EpiQuik Total Histone H3 Acetylation Assay Kit (**G.**, n = 4) and immunoblot analysis using anti-Acetyllysine antibody (**H.**, n = 4) in HEK293T cells transfected with empty vector or vector with GM7239 ORF coupled to HA tag inserted. Complete blots are shown in supplementary figures. Boxplots represent the median and interquartile range (IQR); whiskers mark 1.5x the IQR; data beyond 1.5x the IQR are plotted as individual points. Statistics by t test: * is p-value<0.05, ** is p-value < 0.01, *** is p-value < 0.001. Grey dots represent the value for each individual replicate.

The sharp increase in transcription at *Dock4* locus occurs on the opposite strand to *Dock4* gene (Figure 6A). Multiple predicted pseudogenes, including *Gm7239*, are detectable in this 50 kb region that is highly transcribed with aging (Figure 6A). Gm7239 was recently reported to encode a microprotein ^41^. We observed that its predicted sequence is nearly identical to the C-terminal domain of mouse SET isoform 1 (RefSeq: NP_076360.1)(Figures 6B, 6C). To eliminate the possibility that the sharp increase in transcription at *Gm7239* could be due to contamination by reads from the *SET* coding gene, we quantified the transcript levels of *Gm7239* and *SET* genes using *de novo* transcript analysis from 3-, 12-, 18- and 24-month old mouse retina (Supplementary Figures 8A and B). The transcripts of *Gm7239*, but not *SET*, increase with aging (Supplementary Figure 8C). *Gm7239* is actively translated in mouse skin, and its deletion leads to major transcriptional reprogramming ^41^. This small microprotein encodes the HAT inhibitory domain of SET as well as its histone and DNA binding domain and appears devoid of regulatory sequences present in the full-length SET protein (Figure 6C). Thus, we predicted that *Gm7239* microprotein expression in aging may lead to a decrease in histone acetylation. Consistently, the global levels of H3 acetylation (Figure 6D) and H3K27ac (Figure 6E; Supplementary Figure 9A) significantly decrease through aging. At the last time point in aging, the level of H3K18Ac increases, pointing to potential compensation mechanisms (Supplementary Figures 9B and C).

To assess whether *Gm7239* microprotein encodes a functional HAT inhibitor, we cloned the *Gm7239* small ORF in a mammalian expression vector with a C-terminal tag. Transient transfection of this construct or corresponding empty vector into HEK293T cells, followed by immunoblot analysis, confirm the expression of the tagged protein of approximately 20 kDa in the transfected cells (Figure 6F; Supplementary Figure 9D). Correspondingly, we observe a significant decrease in total H3 acetylation by ELISA (Figure 6G) and pan-global histone lysine acetylation, as detected by immunoblot analysis of histones purified from cells transfected with *Gm7239* compared to the empty vector (Figure 6H; Supplementary Figure 9E). These results strongly argue for the role of the *Gm7239* in inhibiting histone acetylation and suggest that age-induced expression of the active protein domains encoded by microproteins like *Gm7239* could represent a potential mechanism of chromatin remodeling with advanced age.

### Transcriptional changes correlate with altered chromatin landscape at AMD genes

**Figure 7:**
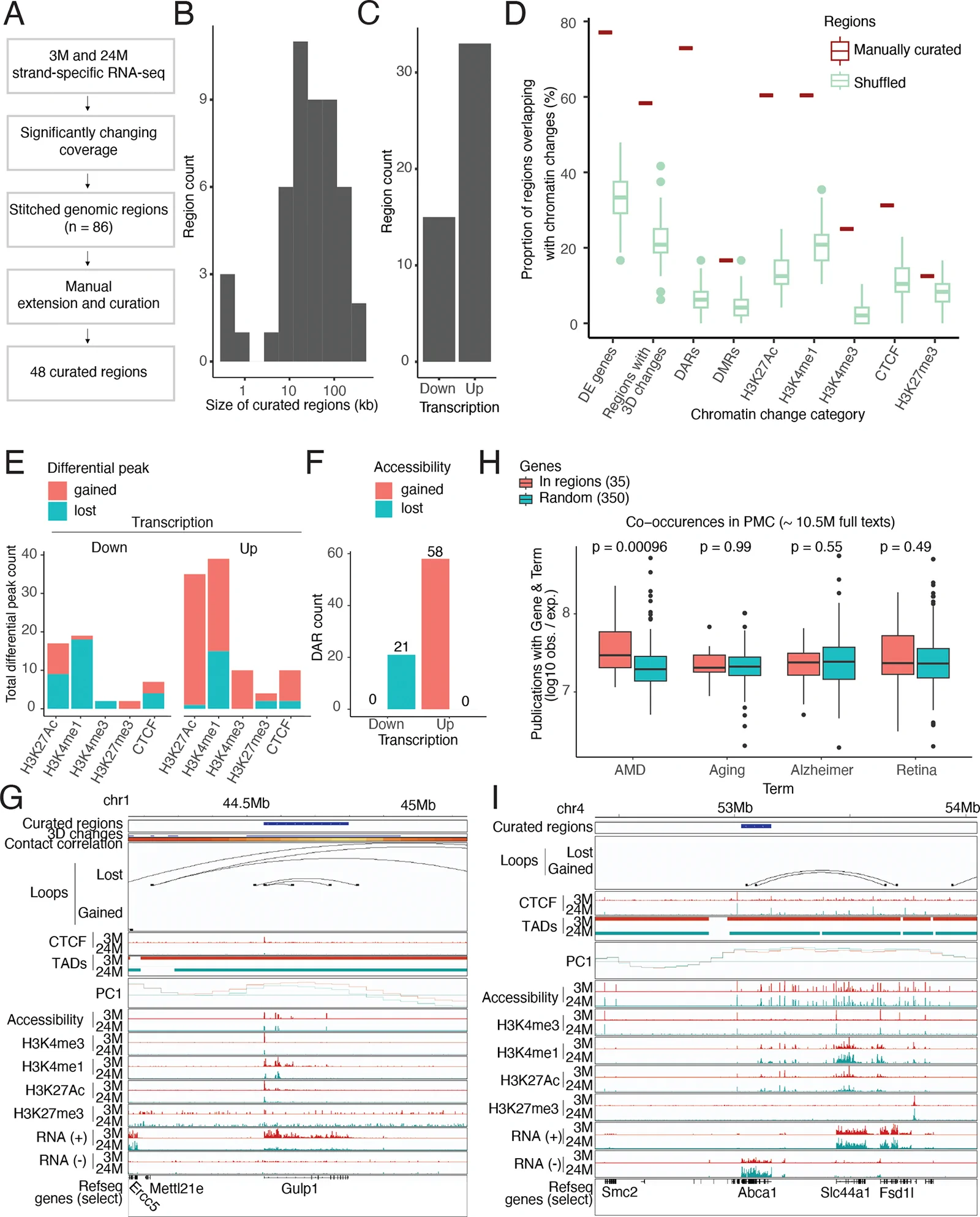
Most significant changes in transcription are associated with change in chromatin landscape. **A.** Schematics of the pipeline to identify and manually curate regions with significant transcription changes. **B.** Size distribution of regions manually curated for transcription changes. **C.** Count of manually curated regions for transcription changes down-regulated (n=15) and up-regulated (n=33). **D.** Proportion of regions identified in **A.** (dark red) or 100 sets of shuffled regions (light green) overlapping with changes in chromatin landscape (differential gene expression – “DE genes” –, 3D conformation – “Regions with 3D changes” –, accessibility – “DARs” –, DNA methylation – “DMRs” – and differential histone marks / CTCF binding). **E.** Count of regions differentially enriched for histone mark or bound by CTCF overlapping regions identified in **A.** depending on their transcription change. **F.** Count of differentially accessible regions (DARs) overlapping regions identified in A. depending on transcription change. **G.** Overview of the *Gulp1* locus on chr1 (mm10 coordinates). Tracks represent from top to bottom: regions identified in **A.**, regions with 3D changes, global pattern correlation between 3 and 24 months (yellow for low correlation, red for high correlation), chromatin loops, CTCF coverage, TADs, compartments (PC1), chromatin accessibility (log scale), H3K4me3, H3K4me1, H3K27ac and H3K27me3 coverages and transcription on + strand and − strand at 3- (red) or 24-months old (blue), and a selection of RefSeq genes. **H.** Observed over expected ratios of publications number with co-occurrence of Refseq gene names overlapping the regions identified in **A.** (red) or 350 random gene names (blue) and terms among “AMD” or “Age-related macular degeneration”, “Aging”, “Alzheimer” or “Retina”. **I.** Overview of the *Abca1* locus on chr4. Tracks represent from top to bottom: curated regions identified in **A.**, chromatin loops, CTCF coverage, TADs, compartments (PC1), chromatin accessibility (log scale), H3K4me3, H3K4me1, H3K27ac and H3K27me3 coverages, transcription on + strand and − strand at 3- (red) or 24-months old (blue), and a selection of RefSeq genes. Boxplots represent the median and interquartile range (IQR); whiskers mark 1.5x the IQR; data beyond 1.5x the IQR are plotted as individual points. Statistics by t test.

We then computed additional genomic regions with strong changes in transcription independently of any gene annotation (Figure 7A). After manual curation, we identified 48 large genomic regions (10–100 kb) with significant changes in local transcription during aging (Figure 7B, Supplementary Table 2). Most of these regions are upregulated (33 over 48, Figure 7C) and enriched for DE genes, 3D changes (mostly altered chromatin looping and interaction pattern correlation), DARs, changes in active histone marks and CTCF binding (Figure 7D, Supplementary Figure 10A). We then examined the correlation between altered transcription and changes of chromatin marks at differentially bound loci. Globally, the regions with decreased transcription tend to lose active chromatin marks and those with enhanced expression generally gain these marks (Figure 7E). This trend is even more prominent when looking at differentially accessible loci. Indeed, regions with decreased transcription overlap exclusively with lost DARs while regions with increased transcription overlap exclusively with gained DARs (Figure 7F). For example, the decrease in both RNA and protein levels of *Gulp1* gene is associated with diminution of interaction pattern correlation and compartment score (PC1), loss of several chromatin loops, accessible sites and active histone marks in aging (Figure 7G, Supplementary Figure 10B). Transcription factor motifs enriched at gained or lost accessible regions of these loci reveal several potential drivers of transcriptional changes, including RARG, PO6F2 and LHX2 for up-regulation and ZFP57 and SOLH2 for down-regulation (Supplementary Figure 10C).

Thirty of the 48 manually curated regions overlap with known genes on the same strand (35 genes total, Supplementary Table 2) and two are within 10 kb of a known gene on the same strand, indicating an alternative promoter (*Sde2*) or a change in transcription termination (*Prph2*). Several of these genes have previously been linked to retinopathies, AMD, and/or Alzheimer’s disease: these include *Gulp1* ^42^, *Mylk* ^43^, *Myo10* ^44^, *Nxnl2* ^45^, *Prph2* ^46^, *Rho* ^47^, *Rorb* ^48^, *Ttyh2* ^49^, *Wdr31* ^50^, *Abca1* ^51^, *Fndc1* ^52^, *Galntl6* ^53,54^, *Glb1l3* ^55^, *Hdc* ^56^, *Mef2c* ^57,58^, *Rdh1* and *Rdh9* ^59^. We then performed a literature co-occurrence analysis to evaluate whether the identified genes are preferentially represented with aging-associated diseases and uncover a significant enrichment of co-occurrence with “AMD,” but not with “Aging,” “Alzheimer,” or “Retina” compared to a set of 350 random genes (Figures 7H and I). Notably, sixteen of the genomic regions that include the *GM7239*-microprotein identified in this study do not overlap with any known gene. As an example, a well-delimited region within a cluster of zinc-fingers protein coding genes is strongly expressed in aging with gain of chromatin loops, increased accessibility, and active histone marks (Supplementary Figure 10D).

We therefore conclude that chromatin remodeling results in de-repression of selected silenced genes or pseudogenes in the aging rod photoreceptors.

## Discussion

Cell type-specific 3D genome organization is a key regulator of gene expression and consequently biological processes, and its disruption is associated with genomic instability and aberrant transcription ^60,61^. In human fibroblasts, replicative cellular senescence leads to an increase in short-range interactions associated with global compaction of the chromatin ^40^. Aging is associated with a gain of long-range interactions as well as a decrease of TAD boundary insulation in mouse satellite cells ^62^ and local 3D chromatin changes in muscle stem cells ^63^. We currently have limited understanding of chromatin remodeling and epigenetic changes during neuronal aging as well as their impact on gene expression and cellular function ^64,65^. Two different single cell studies of 3D chromatin interactions in the human brain have reported a cell population enriched for long-range interactions that correlate with age. In the first study, the correlation is with the donor age ^66^, whereas in the second, it is with an inferred cellular age based on marker genes but not with the donor age ^67^. We note that the enrichment for long-range interactions in single cells correlates with chromosome condensation in mitotic cells ^68^. Here, we demonstrate comprehensive age-associated adaptations in the genomic architecture of a sensory neuron, the rod photoreceptor, and correlate these to changes in expression of genes, which in turn are proxy of functional deviations observed with age.

Studies focused on aging are often limited by high variability between samples, especially in humans. Our focus on the rod photoreceptor aging in mice has demonstrated highly reproducible alterations in chromatin landscape, associated with global nuclear changes as well as local effects on specific genes. In addition, while aging studies often rely on bulk sequencing, we exploited the specific inverted architecture of rods and were able to physically validate molecular changes in 3D genome topology. This integrated framework, combining genomics with structural microscopy, provides a robust template for dissecting the physical basis of epigenetic dysregulation. Rod chromatin undergoes increased compaction between 3- and 24-month mice, concomitant with a gradual decrease of global histone H3 acetylation, and reduced H3K27ac levels. In addition, local changes in 3D chromatin architecture are strongly enriched for active chromatin regions, usually characterized by histone acetylation. Loss of H3 acetylation in aging could be a leading cause of the global chromatin compaction. The discrepancies between changes of short- and long-range interactions in different aging studies ^40,62,66,67^ could be explained by dynamics of histone acetylation in different cell types. Decreased acetylation in aging has also been observed in other tissues ^69,70^, but its causation is still unclear. We and others have shown that aging is associated with metabolic changes including decreased glycolysis ^4,29^. Given that glycolysis also contributes to the cellular acetyl-CoA pool for histone acetylation, a reduced glycolytic flux could also contribute to global decrease of H3 acetylation. Gm7239 is particularly intriguing since it illustrates how conventional pseudogene annotations may overlook functional components of the proteome. Various studies including an *in vivo* CRISPR screen ^41,71^ supports its translation as a biologically relevant nuclear microprotein, and our findings suggest its role as a functional histone acetyltransferase inhibitor. We propose that the accumulation of this inhibitory protein directly affects histone acetylation levels, acting synergistically with metabolic changes to drive the global chromatin compaction in aging rod photoreceptors. Further investigations on Gm7239 and other age-associated microproteins could yield new insights into the links between chromatin re-modeling and de novo gene expression during aging-associated cellular adaptations that may contribute to disease susceptibility.

The distribution of H3K27ac signal on chromatin remains very similar between 3- and 24-months. Unfortunately, we could not obtain reliable CUT&Tag spike-in signal to confidently identify a global decrease in H3K27ac. However, we detect clear local changes in H3K27ac with aging as well as other marks. Notably, these changes as well as changes in 3D chromatin structures and DNA methylation do not fully overlap although they cluster in the same regions. Single locus observation shows that changes in histone marks, accessibility, CTCF binding, chromatin loops, TADs or compartment form a global picture explaining the alteration of expression at the observed locus, as we show here in detail for *Myo10* or *Dock4* loci. This could potentially be the primary reason for the incomplete overlap between changes: only part of the chromatin marks may overlap in each locus. The complexity arises when trying to extract general rules governing genome-wide age-related changes. The mechanism associated to transcription alteration in aging seems to be locus-dependent: changes in CREs (gain / loss of histone marks), gain or loss of a CRE-promoter loop, associated or not with a gain / loss of chromatin accessibility or CTCF binding, change of chromatin compartment, or sometimes, simple change of histone marks at the promoter of the altered gene. In addition, our expression data are based on RNA-seq data, which means that alterations of RNA stability may mask or inflate alterations of transcription we infer from these data. The alterations we observe in aging likely vary in terms of underlying mechanism but are reproducible across biological replicates, pointing to active processes of chromatin regulation rather than epigenetic drift.

Aberrant de-repression of pseudo-genes or other genomic elements such as LINEs is a phenomenon associated with aging and age-related diseases ^72^. Such alterations can have drastic consequences for the cells. In addition to generate genome instability, these genomic elements can contain small ORFs, encoding for functional active domains as we show here for the HAT inhibitor encoded by *Gm7239*. We have identified and manually curated regions with the most significant transcription alterations, independently of any known gene annotation. We uncover a large fraction of non-annotated altered regions (18 out of 48) that are mostly upregulated during aging. These regions span dozens of kb and contain a plethora of potential ORFs. In addition, the remaining top regions with transcriptional alterations overlap with genes enriched for co-occurrences with AMD in scientific literature. We realize that co-occurrence does not capture the nature of the association between each gene and AMD and that could represent mechanistic change in expression, association to genetic variant, or simple mention of both terms in the same publication without any specific link. We also note that mice do not naturally develop AMD. Therefore, it would be challenging to elucidate how altered chromatin landscape we detect in aging mouse retina relates to AMD.

To further evaluate the potential relevance of these genomic regions, we examined previously-published human retinal expression and DNA-methylation quantitative trait locus datasets ^21^. *IMMP2L* expression was associated with rs37672, and two significant mQTL associations were identified at this locus: rs17158169–cg09628812 and rs1540856–cg14182954. The latter association was classified as retina specific. At the *DPP10* locus, rs2420815 was associated with gene expression, whereas six significant mQTL associations were detected across the locus. Notably, the association involving cg16948172 was also classified as retina specific. These findings indicate that *IMMP2L* and *DPP10* are subject to genetic regulation at both the transcriptional and DNA-methylation levels in the human retina. Thus, some regulatory features at these loci are likely tissue dependent. One hypothesis would be that aging-associated chromatin changes exacerbate rod vulnerability to metabolic and oxidative stress and contribute to AMD pathology in humans. Further studies using human retina samples, especially rods from different spatial regions, may provide mechanistic insights into the role of aging in AMD causation.

Our study raises new questions related to potential mechanisms of aging. Is the global chromatin architecture and H3 acetylation of human rods impacted by aging in the same way as in mouse? Is de-repression of the non-annotated regions also observed in aging human retina? How does chromatin remodeling contribute to age-related retinal diseases? We are optimistic that future studies translating these findings to human retina, and probably in other tissues, will help discover ways to reverse chromatin and epigenome remodeling to prevent age-associated disease phenotypes.

## Materials and Methods

### Mice

All procedures involving mice were approved by the National Eye Institute Animal Care and Use Committee (ASP #650). Mice were kept in 12 hr light and dark cycle and fed ad libitum at NEI animal care facility. C57BL/6J mice expressing eGFP under the control of the *Nrl* promoter (Nrlp-EGFP mice) ^28^ were used to purify rod photoreceptors by flow sorting. Wild-type aging mice (C57BL/6J) were obtained from Jackson Laboratory (www.jax.orgwww.jax.org) and National Institute of Aging resource.

### Rod purification and cross-linking

Rod photoreceptors were purified using the papain dissociation method, adapted from a previous study ^73^ with slight modifications for the Hi-C experiment. Briefly, 2–4 retinas were dissected and processed for papain dissociation in a 5 ml polypropylene tube. After dissociation, cells were fixed for 10 minutes in 2% PFA, followed by quenching with 1M glycine to a final concentration of 125 mM for 5 min. Two washes with ice-cold 1X PBS were done, and samples were processed for flow cytometry with a 100 μm nozzle to isolate GFP-positive cells. For CUT&Tag, ATAC-seq and RNA-seq after dissociation, cells were washed with ice cold 1X PBS and sorted without fixation.

### RNA-seq analysis

We used our previously published rod RNA-seq data from 3- and 24-month retina (4 replicates per group ^29^). Windows with a difference of RNA coverage were called using SwitchRT (github.com/ClaireMarchal/SwitchRT, q-value < 0.2). Windows within 10 kb were stitched together using bedtools ^74^ and then manually curated by plotting strand-specific RNA for each group of age in IGV. Manual curation involved the evaluation of each region, filtering for regions with a clear reproducible gradual change of transcription across age groups and redefining the boundaries of each region to include the entire transcribed locus.

### Hi-C

Rods were sorted from 3- and 24-month mouse retinas (5 biological replicates per age group). Hi-C libraries were generated using Arima HiC+ kit and sequenced on NextSeq 2000 (Illumina, CA, USA) at a read length of 150 base pairs, as described ^36^. Hi-C reads from 5 biological replicates per age group (4 replicates with high resolution and 1 having lower resolution) were processed using HiCUP ^75^ and converted to HOMER tag directories, .hic files and .cool files, using HOMER ^76^, juicer ^77^ and cooler ^78^, respectively. Resolutions for each replicate as well as of the merged replicates were manually computed on chr1, following principles previously described ^79^. Briefly, read coverage from valid interactions on chr1 was computed for each replicate / merged replicates using bedtools with window sizes increasing of 100 bp until the 20^th^ percentile reaches 1000 or more. Stratum-adjusted Correlation Coefficient (SCC) was computed on chr1 using hicrep ^80^, at 50 kb resolution, with a smoothing factor of 5. Interaction distance distribution was computed using R in each of the 5 biological replicate, using 10 M random valid interactions for each replicate. Compartmentalization and saddle plot were generated for each of the 5 replicates using cooltools ^81^. Compartments were computed using HOMER in each of the 5 replicates. TADs were computed in each of the 4 high resolution replicates using domaincaller ^82^ at 10 kb resolution and in the merged replicates to plot TADs in examples profiles. Loops were called using Mustache at 5kb resolution ^83^. Changes in compartments were computed using HOMER using the 5 replicates. Changes in interaction pattern were computed using HOMER on the merged replicates. These changes in interaction patterns are computed by comparing the interaction profile for a given locus in the 3-month merged samples to the interaction profile from the same locus in the 24-month merged samples. For each chromatin locus, it results in a correlation value between 3- and 24-month. Here, a high value (close to 1) means the samples are very similar, whereas a low value (0 to 0.8) indicates differences in the samples for interactions at a given locus. Changes in loops were computed using Mustache at 5kb resolution. Changes in TADs boundaries were computed using bedtools ^74^. Diamond insulation scores were computed using cooltools using 10 kb resolution with 100 kb windows and default options in each of the 4 high-resolution replicates. Aggregate peaks analysis (APA) was computed on the merged replicates and on the 4 high resolution replicates using juicer. Contact maps were extracted from the merged 5 biological replicates using HOMER and plotted using R package ggplot2.

### ATAC-seq

ATAC-seq libraries were prepared from purified rods from 3-, 12-, 18- and 24-month mouse retina (4 biological replicates per age), as described ^36,84^. The analysis was performed using ENCODE ATAC-seq pipeline (atac_dnase_pipeline). Accessible motif analysis used accessible footprints within differentially accessible regions, as described ^36^.

### CUT&Tag

CUT&Tag was performed on 3-month and 24-month rods using CUT&Tag-IT Assay Kit, Anti-Rabbit (Active Motif, cat.no. 53160) with slight modifications, as reported earlier ^85^. Antibodies details are as follows: H3K27ac (Rabbit, cat.no. ab4729, Abcam, Cambridge, UK), H3K27me3 (Rabbit, cat.no. ab195477, Abcam, Cambridge, UK), H3K4me3 (Rabbit, cat.no. ab8580, Abcam, Cambridge, UK), H3K4me1(Rabbit, cat.no. ab8895, Abcam, Cambridge, UK) and CTCF (Epicypher, CUTANA^™^ CUT&RUN, SKU: 13–2014). All antibodies were used at a dilution of 1:100.

Sequenced libraries were locally mapped on a chimeric genome, including mouse (mm10), S. cerevisiae (GCA_000146045.2) and E. coli (GCA_000005845.2) genomes, using bowtie2 ^86^. Mapped reads were filtered based on quality (q > 20) and duplicates were removed using samtools ^87^. Reads mapping on mm10 genome, excluding mm10 blacklisted regions (github.com/Boyle-Lab/Blacklist/blob/master/lists/Blacklist_v1/mm10-blacklist.bed.gz), were kept for further analysis using samtools. Samples with a ratio of same over different strand reads (computed using HOMER ^76^) over 0.5 were selected for further analysis. For each mark, samples were sub-sampled to the number of reads of the smaller sample from the same mark, using samtools. Peak calling was performed on subsampled samples using macs2 ^88^ with options: “-g 2700000000 -f BAMPE --llocal 50000 --broad --broad-cutoff 0.1 -q 0.01 -B --SPMR --keep-dup all”. Overlapping peaks between replicates were computed using iterative peak overlap, with options “--spm 5 --rule "(n+1)/2" --extend 10” ^89^. Sub-sampled replicates were merged using samtools. Sub-sampled replicates and merged samples were then analyzed using HOMER, bedtools ^74^ and R. Bigwig files were generated using BamCoverage ^90^, with a normalization in CPM. Figures have been plotted using R and IGV ^91^.

### Microscopy measurements of rod photoreceptors

Eyes from C57BL/6J mice (3- and 24- months old) were enucleated and fixed in 4% (w/v) paraformaldehyde for 1 hour at room temperature. Following fixation, the eyes were washed in PBS thrice for 10 minutes each. The eyes were then consecutively incubated in 10% and 20% sucrose for one hour each. 30% sucrose was then added to the samples for overnight incubation at 4°C. After dissection, the eye cups containing only the retina were embedded in optimal cutting temperature (OCT) medium. 10 μm retinal sections were cut on SuperFrost Plus slides using a Leica CM1850 cryostat (Wetzlar, Germany). The sections were then incubated in blocking buffer (5% normal donkey serum, 0.5% BSA, 0.2% Tween-20 and 0.05% sodium azide in PBS (pH 7.3)) for 3 hours at room temperature. Overnight incubation of the retinal sections at 4°C was done with the primary antibodies and 1 μg/mL of DAPI. The primary antibodies were purchased as follows: anti-NRL (3:400, cat. no. AF2945, R&D Systems), anti-H3K27ac (3:100, cat.no. ab4729, Abcam) and anti-H3K9me3 (3:100, cat. no. 39062, Active Motif). Secondary antibodies were incubated overnight at 4°C. The following secondary antibodies were used: anti-Goat Alexa Fluor 594 (1:150, Invitrogen, cat.no. A-11058) and nanobody-anti-rabbit STAR RED (1:100, cat.no. STRED-1010–30UG, Abberior). Sections were mounted using Prolong Glass (Invitrogen, P36980) and #1.5 coverslips (Avantor, VWR, cat. no. 48393–230). Images were acquired at 63x magnification in with a Leica TCS SP8 in lightning mode. Huygens (Professional) software package was used to deconvolve the images.

### Nuclei surface area over volume ratios and sphericity analysis

Quantitative analysis of the acquired images was performed for DAPI, NRL, H3K9me3 and H3K27ac signals using Aivia AI image analysis software (Leica Microsystems, version 15). Aivia AI image analysis software (Leica Microsystems) was used to compute surface area over volume ratios and sphericity from nuclei. The data was filtered for nuclei over 1 μm from any border (x, y and z) and with a volume between 20 and 40 μm^3^ (mouse rod photoreceptors have a volume of ~ 30 μm^3 92,93^. Nuclei surface area over volume ratios and sphericity were plotted by calculating for each biological replicate the average nuclei surface area over volume ratios and sphericity across all filtered nuclei. The mean of the 3 biological replicates and the standard error of the mean were then computed for each age group and plotted.

### Immunofluorescence signal distribution analysis

Coordinates of filtered nuclei (see “Nuclei surface area over volume ratios and sphericity analysis” part) were then used to analyze the DAPI, H3K9me3, H3K27ac and NRL signal distribution relative to the nuclei center. To do so, the raw data were extracted by converting the .lif file into a .tif file for each condition / signal / z stack using bftools ^87^, then converting the .tif file into a text file using readTIFF function from R package tiff. Signal intensity for each pixel around each filtered nucleus center has been then extracted using awk. These data were then analyzed with R: First the 3D distance to the nucleus center of mass was computed for each pixel. Then pixels were grouped by distance category from each nucleus center using 3D windows of 0.1 μm thickness. The median intensity for each signal within each window was computed for each nucleus. The nucleus boundary position relative to the center was estimated in each case by fitting the DAPI median intensity relative to the distance to the nucleus center into a polynomial function and using this function to extract the position of the minimum intensity, marking the nucleus limit. NRL, H3K9me3 and H3K27ac positions relative to the nucleus center were extracted by computing the position of the mark peak relative to the nucleus center (see Figure 2L). The mean of these values was computed from all the nuclei of each biological replicate and the mean and standard error of the mean from the three replicates were computed and plotted.

### Histone extraction and H3 acetylation assay

Histones were purified from flash-frozen whole retina tissues obtained from four biological replicate of C57BL/6J mice of different age groups (3-month, 9–12 month, 22–24 month) using Abcam Histone Extraction Kit (cat. no. ab221031). The purified histones were quantified using BCA assay and assessed through western blot using quantified Revert^™^ Total Protein Stains. H3 Acetylation Assay was performed using EpiQuik Global histone H3 acetylation assay kit (Cat. no. P-4008–48). Protein concentration was adjusted to 200 ng/μl. OD values, proportional to histone H3 acetylation levels, were plotted.

### Immunoblotting

Histone proteins were separated on 4–15% SDS-PAGE and transferred onto LF polyvinylidene fluoride (PVDF) membranes (Millipore). Total protein on the membranes was visualized using the LI-COR Revert^™^ Total Protein Stains according to the manufacturer’s instructions. Membranes were then blocked with LI-COR (TBS) Blocking Buffer and incubated overnight at 4 °C with the following primary antibodies: anti-H3K27ac (rabbit, cat.no. ab4729, Abcam,) anti-H3K18Ac (rabbit, cat.no. ab1191, Abcam), and anti-Acetyllysine antibody (mouse mAb, cat. no. PTM-102). Membranes were then washed four times with TBST and incubated with LI-COR-488 secondary antibodies at room temperature for 1hr. The immunoreactive products were detected using Licor Odyssey Imaging System. ImageStudio was used to perform quantitative analysis of western blot results as the ratio of the band intensities of target histone marks to the corresponding band intensities of reference total protein.

### Gm7239 ORF cloning and transfection

A synthetic DNA fragment, encoding the Gm7239 open reading frame (ORF), was synthesized by Genewiz (Azenta). The lyophilized DNA (2 μg total) was reconstituted in 20 μL of nuclease-free water to a final concentration of 100 ng/μL. The DNA was cloned into the mammalian expression vector pcDNA4 (Thermo Fisher Scientific), which contains a CMV promoter and a C-terminal HA epitope tag. For cloning, both the vector and insert were digested with EcoRI-HF and XhoI restriction enzymes (New England Biolabs) in CutSmart buffer. Each 50 μL reaction contained 1 μg of DNA, 1 μL of each enzyme, 5 μL of 10x CutSmart buffer, and nuclease-free water to volume. The mixture was incubated at 37 °C for 1 h and heat-inactivated at 65 °C for 20 min. The digested DNA fragments were separated by agarose gel electrophoresis, and the appropriate bands were purified using the Gel Extraction Kit (Qiagen). Purified vector and insert were ligated at a 3:1 insert-to-vector molar ratio using T4 DNA ligase (NEB) for 1 h at room temperature. The ligation products were transformed into E. *coli* DH5α competent cells and positive clones were identified by restriction enzyme analysis as well as Sanger sequencing. Plasmid DNA was performed using QIAprep Spin Miniprep Kit (QIAGEN) according to the manufacturer’s protocol.

For transient expression, HEK293T cells were cultured in DMEM supplemented with 10% fetal bovine serum and 1% penicillin–streptomycin at 37 °C in 5% CO_2_. Cells were seeded in six-well plates one day prior to transfection to achieve 70–80% confluence. Transfections were performed using X-tremeGENE 9 DNA Transfection Reagent (Roche, cat. no. 06365787001) according to the manufacturer’s instructions. Briefly, 1 μg of plasmid DNA (pcDNA4-Gm7239-HA or empty vector control) was diluted in 100 μL Opti-MEM medium and combined with 3 μL of X-tremeGENE 9 reagent (3:1 reagent-to-DNA ratio, v/w). After incubation for 15 min at room temperature, the complexes were added dropwise to the cells. Cells were harvested 48 hr post-transfection for Western-blot and ELISA analysis. Expression of the HA-tagged Gm7239 protein was confirmed by Western-blot, showing a distinct ~20 kDa band in transfected samples.

### Targeted mass-spectrometry

#### Sample Processing:

Tryptic peptide FLGSTEVEQPK corresponding to GULP1 was obtained as SpikeTide L peptides from JPT Peptide Technologies. Mouse retinas were lysed in 8M urea. Equal amounts of protein from each sample were reduced with 10 mM dithiothreitol (DTT) and alkylated with 20 mM iodoacetamide (IAA). Samples were then diluted with 50 mM triethylammonium bicarbonate (TEAB) to a final urea concentration of <1M. Proteins were digested overnight at 37°C using TPCK-treated trypsin/LyC (1:25 ratio). The resulting peptides were desalted using a C18 stage tip and lyophilized.

#### Parallel Reaction Monitoring (PRM) data Acquisition:

Peptide samples were reconstituted in 0.1% formic acid and quantified using a BCA peptide kit. A synthetic peptide (1 pmol) was spiked into each sample as an internal standard prior to analysis. Peptides were separated on a C18 column using an UltiMate 3000 RSLCnano system (Thermo Fisher Scientific) coupled to a Q Exactive Mass Spectrometer (Thermo Fisher Scientific). The mobile phases consisted of (A) 0.1% formic acid in water and (B) 0.1% formic acid in acetonitrile. The peptides were resolved on a reverse phase column using flow rate of 50ml/min over a gradient of 110 min and a total run time of 140 min. Mass spectrometry data were acquired in Data-Independent Acquisition (DIA) mode with 4m/z isolation window. The Q Exactive mass spectrometer was configured to run with an inclusion list containing the mass-to-charge ratio (m/z) and charge state (z) for the targeted peptide.

#### PRM Data Analysis

PRM data were processed and analyzed using Skyline software, version 3.5 (MacLean et al., 2010). Extracted ion chromatograms (XICs) were generated for all corresponding transitions of each target peptide. Each transition was manually inspected, and Savitzky-Golay smoothing was applied to the peaks. The composite peak area for each peptide was calculated by summing the area under the curve (AUC) of its transitions. The light-to-heavy peptide ratio was then calculated to obtain normalized area for quantification.

$$Normalized\;Area\;=\frac{AUC_{\mathrm{light}}}{AUC_{\mathrm{heavy}}}$$

Where:

- $AUC_{\text{light}}$ is the area under the curve for the endogenous (light) peptide.
- $AUC_{\text{heavy}}$ is the area under the curve for the synthetic (heavy) spiked-in peptide.

### Gene – Phenotype co-occurrences in PubMed Central

We measured the co-occurrences of each gene name and phenotype pair in full text papers available in Pubmed Central to assess whether the pair association in literature is only due to chance of a random gene name being associated to a phenotype among the plethora of scientific publications. We computed the observed / expected ratio of co-occurrences of the 35 genes identified and the terms “Age-related macular degeneration OR AMD”, “Aging”, “Alzheimer” and “Retina”, using 350 randomly picked genes as negative control. The observed values for each pair were computed using NCBI esearch tool. The expected values for each pair were assessed by computing the number of publications containing each term of the pair separately using NCBI esearch tool and by estimating the total number of publications on Pubmed Central at the time of the analysis at 10.5 million. Genes were eliminated if no publication existed on Pubmed Central at the time of the analysis.

## Supplementary Material

Supplementary Files

This is a list of supplementary files associated with this preprint. Click to download.

• SupplementaryMaterial08052026.pdf

## Data Availability

Sequencing data produced in this study are available on GEO portal under accession GSE334822. To review, please go to: https://www.ncbi.nlm.nih.gov/geo/query/acc.cgi?acc=GSE334822 Enter token gtenwgqmdtmrpkp into the box Raw microscopy data are available on Zenodo at the following URL: https://doi.org/10.5281/zenodo.20633253

## Code availability

Custom scripts used in this study can be accessed on github repository: github.com/NEI-NNRL/2026_Mouse_Retina_Aging_HiC

## References

[1] Gladyshev V.N. Disagreement on foundational principles of biological aging. PNAS Nexus 3, pgae499 (2024). 39660064 10.1093/pnasnexus/pgae499 PMC11630784

[2] Booth L.N. & Brunet A. The Aging Epigenome. Mol Cell 62, 728–44 (2016). 27259204 10.1016/j.molcel.2016.05.013 PMC4917370

[3] Melzer D., Pilling L.C. & Ferrucci L. The genetics of human ageing. Nat Rev Genet 21, 88–101 (2020). 31690828 10.1038/s41576-019-0183-6 PMC9934000

[4] Lopez-Otin C., Blasco M.A., Partridge L., Serrano M. & Kroemer G. Hallmarks of aging: An expanding universe. Cell 186, 243–278 (2023). 36599349 10.1016/j.cell.2022.11.001

[5] Shen X. Nonlinear dynamics of multi-omics profiles during human aging. Nat Aging 4, 1619–1634 (2024). 39143318 10.1038/s43587-024-00692-2 PMC11564093

[6] Hou Y. Ageing as a risk factor for neurodegenerative disease. Nat Rev Neurol 15, 565–581 (2019). 31501588 10.1038/s41582-019-0244-7

[7] Shenhar B. Heritability of intrinsic human life span is about 50% when confounding factors are addressed. Science 391, 504–510 (2026). 41610249 10.1126/science.adz1187

[8] Zhang W., Qu J., Liu G.H. & Belmonte J.C.I. The ageing epigenome and its rejuvenation. Nat Rev Mol Cell Biol 21, 137–150 (2020). 32020082 10.1038/s41580-019-0204-5

[9] Seale K., Horvath S., Teschendorff A., Eynon N. & Voisin S. Making sense of the ageing methylome. Nat Rev Genet 23, 585–605 (2022). 35501397 10.1038/s41576-022-00477-6

[10] Mondal A.K., Gaur M., Advani J. & Swaroop A. Epigenome-metabolism nexus in the retina: implications for aging and disease. Trends Genet 40, 718–729 (2024). 38782642 10.1016/j.tig.2024.04.012 PMC11303112

[11] Sahu V. & Lu C. Metabolism-driven chromatin dynamics: Molecular principles and technological advances. Mol Cell 85, 262–275 (2025). 39824167 10.1016/j.molcel.2024.12.012 PMC11750176

[12] Schmauck-Medina T. Dietary restriction in aging and longevity. Nat Aging 6, 485–505 (2026). 41792328 10.1038/s43587-026-01091-5

[13] Lautrup S., Sinclair D.A., Mattson M.P. & Fang E.F. NAD(+) in Brain Aging and Neurodegenerative Disorders. Cell Metab 30, 630–655 (2019). 31577933 10.1016/j.cmet.2019.09.001 PMC6787556

[14] Pan W.W., Wubben T.J. & Besirli C.G. Photoreceptor metabolic reprogramming: current understanding and therapeutic implications. Commun Biol 4, 245 (2021). 33627778 10.1038/s42003-021-01765-3 PMC7904922

[15] Viegas F.O. & Neuhauss S.C.F. A Metabolic Landscape for Maintaining Retina Integrity and Function. Front Mol Neurosci 14, 656000 (2021). 33935647 10.3389/fnmol.2021.656000 PMC8081888

[16] Hurley J.B. Retina Metabolism and Metabolism in the Pigmented Epithelium: A Busy Intersection. Annu Rev Vis Sci 7, 665–692 (2021). 34102066 10.1146/annurev-vision-100419-115156 PMC8922051

[17] Campello L. Aging of the Retina: Molecular and Metabolic Turbulences and Potential Interventions. Annu Rev Vis Sci 7, 633–664 (2021). 34061570 10.1146/annurev-vision-100419-114940 PMC11375453

[18] Xue Y. & Cepko C.L. Gene Therapies for Retinitis Pigmentosa that Target Glucose Metabolism. Cold Spring Harb Perspect Med 14(2024). 10.1101/cshperspect.a041289 PMC11065158 37460158

[19] Clerin E., Ait-Ali N., Sahel J.A. & Leveillard T. Restoration of Rod-Derived Metabolic and Redox Signaling to Prevent Blindness. Cold Spring Harb Perspect Med 14(2024). 10.1101/cshperspect.a041284 PMC11529851 37848252

[20] Lee J., Han M., Wang K., Butler L.R. & Sinclair D.A. Epigenetic reprogramming for ocular aging and disease: Mechanisms, biomarkers, and the road to the clinic. Prog Retin Eye Res 111, 101442 (2026). 41577329 10.1016/j.preteyeres.2026.101442

[21] Advani J. QTL mapping of human retina DNA methylation identifies 87 gene-epigenome interactions in age-related macular degeneration. Nat Commun 15, 1972 (2024). 38438351 10.1038/s41467-024-46063-8 PMC10912779

[22] Wright A.F., Chakarova C.F., Abd El-Aziz M.M. & Bhattacharya S.S. Photoreceptor degeneration: genetic and mechanistic dissection of a complex trait. Nat Rev Genet 11, 273–84 (2010). 20212494 10.1038/nrg2717

[23] Ingram N.T., Sampath A.P. & Fain G.L. Why are rods more sensitive than cones? J Physiol 594, 5415–26 (2016). 27218707 10.1113/JP272556 PMC5043029

[24] Curcio C.A. & Owsley C. The Science of Rod-Mediated Dark Adaptation, an Outcome Measure for Age-Related Macular Degeneration: The 2025 Proctor Medal Lecture. Invest Ophthalmol Vis Sci 67, 34 (2026). 10.1167/iovs.67.3.34 PMC13001822 41837573

[25] Swaroop A. & Roska B. Revisiting retinal and macular degeneration in the genomics era. Nat Rev Genet (2026). 10.1038/s41576-026-00960-4 PMC13288179 42230802

[26] Subramanian K. Rod nuclear architecture determines contrast transmission of the retina and behavioral sensitivity in mice. Elife 8(2019). 10.7554/eLife.49542 PMC6974353 31825309

[27] Feodorova Y., Falk M., Mirny L.A. & Solovei I. Viewing Nuclear Architecture through the Eyes of Nocturnal Mammals. Trends Cell Biol 30, 276–289 (2020). 31980345 10.1016/j.tcb.2019.12.008 PMC7293902

[28] Akimoto M. Targeting of GFP to newborn rods by Nrl promoter and temporal expression profiling of flow-sorted photoreceptors. Proc Natl Acad Sci U S A 103, 3890–5 (2006). 16505381 10.1073/pnas.0508214103 PMC1383502

[29] Corso-Diaz X. Genome-wide Profiling Identifies DNA Methylation Signatures of Aging in Rod Photoreceptors Associated with Alterations in Energy Metabolism. Cell Rep 31, 107525 (2020). 32320661 10.1016/j.celrep.2020.107525 PMC7228806

[30] Swaroop A., Kim D. & Forrest D. Transcriptional regulation of photoreceptor development and homeostasis in the mammalian retina. Nat Rev Neurosci 11, 563–76 (2010). 20648062 10.1038/nrn2880 PMC11346175

[31] Loell K.J. Transcription factor interactions explain the context-dependent activity of CRX binding sites. PLoS Comput Biol 20, e1011802 (2024). 38227575 10.1371/journal.pcbi.1011802 PMC10817189

[32] Corso Diaz X. Maf-family bZIP transcription factor NRL interacts with RNA-binding proteins and R-loops in retinal photoreceptors. Elife 13(2025). 10.7554/eLife.103259 PMC11884789 40047526

[33] Aldiri I. The Dynamic Epigenetic Landscape of the Retina During Development, Reprogramming, and Tumorigenesis. Neuron 94, 550–568 e10 (2017). 28472656 10.1016/j.neuron.2017.04.022 PMC5508517

[34] Norrie J.L. Nucleome Dynamics during Retinal Development. Neuron 104, 512–528 e11 (2019). 31493975 10.1016/j.neuron.2019.08.002 PMC6842117

[35] Cherry T.J. Mapping the cis-regulatory architecture of the human retina reveals noncoding genetic variation in disease. Proc Natl Acad Sci U S A 117, 9001–9012 (2020). 32265282 10.1073/pnas.1922501117 PMC7183164

[36] Marchal C. High-resolution genome topology of human retina uncovers super enhancer-promoter interactions at tissue-specific and multifactorial disease loci. Nat Commun 13, 5827 (2022). 36207300 10.1038/s41467-022-33427-1 PMC9547065

[37] Qu Z., Batz Z., Singh N., Marchal C. & Swaroop A. Stage-specific dynamic reorganization of genome topology shapes transcriptional neighborhoods in developing human retinal organoids. Cell Rep 42, 113543 (2023). 38048222 10.1016/j.celrep.2023.113543 PMC10790351

[38] D'Haene E. Comparative 3D genome analysis between neural retina and retinal pigment epithelium reveals differential cis-regulatory interactions at retinal disease loci. Genome Biol 25, 123 (2024). 38760655 10.1186/s13059-024-03250-6 PMC11100165

[39] Li J. Single-cell atlas of the transcriptome and chromatin accessibility in the human retina. Nat Genet 58, 418–433 (2026). 41578023 10.1038/s41588-025-02454-1 PMC13050529

[40] Criscione S.W. Reorganization of chromosome architecture in replicative cellular senescence. Sci Adv 2, e1500882 (2016). 26989773 10.1126/sciadv.1500882 PMC4788486

[41] Valdivia-Francia F G.U., Renz PF, Spies D, Ormiston M,, Hyams K, S.C., Dure C, Yigit M, Taborsky D, Khandekar A, & Weber R, Y.H., Ellis SJ, Sendoel A. In vivo single-cell CRISPR screening for microproteins identifies a critical ribosomal component. bioRxiv (2025).

[42] Vivien Chiu W.Y., Koon A.C., Ki Ngo J.C., Edwin Chan H.Y. & Lau K.F. GULP1/CED-6 ameliorates amyloid-beta toxicity in a Drosophila model of Alzheimer's disease. Oncotarget 8, 99274–99283 (2017). 29245900 10.18632/oncotarget.20062 PMC5725091

[43] Velez J.I. Pooling/bootstrap-based GWAS (pbGWAS) identifies new loci modifying the age of onset in PSEN1 p.Glu280Ala Alzheimer's disease. Mol Psychiatry 18, 568–75 (2013). 22710270 10.1038/mp.2012.81 PMC3596442

[44] Chapuis J. Transcriptomic and genetic studies identify IL-33 as a candidate gene for Alzheimer's disease. Mol Psychiatry 14, 1004–16 (2009). 19204726 10.1038/mp.2009.10 PMC2860783

[45] Jaillard C. The metabolic signaling of the nucleoredoxin-like 2 gene supports brain function. Redox Biol 48, 102198 (2021). 34856436 10.1016/j.redox.2021.102198 PMC8640531

[46] Peeters M. PRPH2 mutation update: In silico assessment of 245 reported and 7 novel variants in patients with retinal disease. Hum Mutat 42, 1521–1547 (2021). 34411390 10.1002/humu.24275 PMC9290825

[47] Tang K. Haplotypes of RHO polymorphisms and susceptibility to age-related macular degeneration. Int J Clin Exp Pathol 8, 3174–9 (2015). 26045836 PMC4440145

[48] Leng K. Molecular characterization of selectively vulnerable neurons in Alzheimer's disease. Nat Neurosci 24, 276–287 (2021). 33432193 10.1038/s41593-020-00764-7 PMC7854528

[49] Nalamalapu R.R., Yue M., Stone A.R., Murphy S. & Saha M.S. The tweety Gene Family: From Embryo to Disease. Front Mol Neurosci 14, 672511 (2021). 34262434 10.3389/fnmol.2021.672511 PMC8273234

[50] Cevik S. WDR31 displays functional redundancy with GTPase-activating proteins (GAPs) ELMOD and RP2 in regulating IFT complex and recruiting the BBSome to cilium. Life Sci Alliance 6(2023). 10.26508/lsa.202201844 PMC10200814 37208194

[51] Storti F. Impaired ABCA1/ABCG1-mediated lipid efflux in the mouse retinal pigment epithelium (RPE) leads to retinal degeneration. Elife 8(2019). 10.7554/eLife.45100 PMC6435327 30864945

[52] Dhirachaikulpanich D., Li X., Porter L.F. & Paraoan L. Integrated Microarray and RNAseq Transcriptomic Analysis of Retinal Pigment Epithelium/Choroid in Age-Related Macular Degeneration. Front Cell Dev Biol 8, 808 (2020). 32984320 10.3389/fcell.2020.00808 PMC7480186

[53] Oca A.I. Predictive Biomarkers of Age-Related Macular Degeneration Response to Anti-VEGF Treatment. J Pers Med 11(2021). 10.3390/jpm11121329 PMC8706948 34945801

[54] Tang X. Brain-region-specific, glycosylation-related transcriptomic alterations in Alzheimer's disease. Alzheimer's & Dementia 17(2022).

[55] Le Carre J., Schorderet D.F. & Cottet S. Altered expression of beta-galactosidase-1-like protein 3 (Glb1l3) in the retinal pigment epithelium (RPE)-specific 65-kDa protein knock-out mouse model of Leber's congenital amaurosis. Mol Vis 17, 1287–97 (2011). 21633714 PMC3103743

[56] Satpati A., Neylan T. & Grinberg L.T. Histaminergic neurotransmission in aging and Alzheimer's disease: A review of therapeutic opportunities and gaps. Alzheimers Dement (N Y) 9, e12379 (2023). 37123051 10.1002/trc2.12379 PMC10130560

[57] Escher P., Schorderet D.F. & Cottet S. Altered expression of the transcription factor Mef2c during retinal degeneration in Rpe65−/− mice. Invest Ophthalmol Vis Sci 52, 5933–40 (2011). 21715356 10.1167/iovs.10-6978

[58] Ren J. MEF2C ameliorates learning, memory, and molecular pathological changes in Alzheimer's disease in vivo and in vitro. Acta Biochim Biophys Sin (Shanghai) 54, 77–90 (2022). 35130621 10.3724/abbs.2021012 PMC9909301

[59] Rivolta C. RetiGene, a comprehensive gene atlas for inherited retinal diseases. Am J Hum Genet 112, 2253–2265 (2025). 40961941 10.1016/j.ajhg.2025.08.017 PMC12696501

[60] Fujita Y., Pather S.R., Ming G.L. & Song H. 3D spatial genome organization in the nervous system: From development and plasticity to disease. Neuron 110, 2902–2915 (2022). 35777365 10.1016/j.neuron.2022.06.004 PMC9509413

[61] Sreenivasan V.K.A., Yumiceba V. & Spielmann M. Structural variants in the 3D genome as drivers of disease. Nat Rev Genet 26, 742–760 (2025). 40588575 10.1038/s41576-025-00862-x

[62] Zhao Y. Multiscale 3D genome reorganization during skeletal muscle stem cell lineage progression and aging. Sci Adv 9, eabo1360 (2023). 36800432 10.1126/sciadv.abo1360 PMC9937580

[63] Yang B.A. Three-dimensional chromatin re-organization during muscle stem cell aging. Aging Cell 22, e13789 (2023). 36727578 10.1111/acel.13789 PMC10086523

[64] Yang J.H. Loss of epigenetic information as a cause of mammalian aging. Cell 186, 305–326 e27 (2023). 36638792 10.1016/j.cell.2022.12.027 PMC10166133

[65] Chen X. & Zhu B. Decoding the functions of nuclear speckles in neurodegeneration. Trends Neurosci (2026). 10.1016/j.tins.2026.01.010 PMC13179099 41794640

[66] Prakoso R. Case report: Right atrial appendage hybrid access to bailout a stuck stent from the inferior vena cava of a small child. Front Cardiovasc Med 9, 1084170 (2022). 36776945 10.3389/fcvm.2022.1084170 PMC9912932

[67] Wen X. Single-cell multiplex chromatin and RNA interactions in ageing human brain. Nature 628, 648–656 (2024). 38538789 10.1038/s41586-024-07239-w PMC11023937

[68] Nagano T. Cell-cycle dynamics of chromosomal organization at single-cell resolution. Nature 547, 61–67 (2017). 28682332 10.1038/nature23001 PMC5567812

[69] Dubey S.K. Histone deficiency and hypoacetylation in the aging retinal pigment epithelium. Aging Cell 23, e14108 (2024). 38408164 10.1111/acel.14108 PMC11113634

[70] Signal B. Ageing-Related Changes to H3K4me3, H3K27ac, and H3K27me3 in Purified Mouse Neurons. Cells 13(2024). 10.3390/cells13161393 PMC11353134 39195281

[71] Na Z. Mapping subcellular localizations of unannotated microproteins and alternative proteins with MicroID. Mol Cell 82, 2900–2911 e7 (2022). 35905735 10.1016/j.molcel.2022.06.035 PMC9662605

[72] Li X., Yu H., Li D. & Liu N. LINE-1 transposable element renaissance in aging and age-related diseases. Ageing Res Rev 100, 102440 (2024). 39059477 10.1016/j.arr.2024.102440

[73] Fadl B.R. An optimized protocol for retina single-cell RNA sequencing. Mol Vis 26, 705–717 (2020). 33088174 PMC7553720

[74] Quinlan A.R. BEDTools: The Swiss-Army Tool for Genome Feature Analysis. Curr Protoc Bioinformatics 47, 11 12 1–34 (2014). 10.1002/0471250953.bi1112s47 PMC4213956 25199790

[75] Wingett S. HiCUP: pipeline for mapping and processing Hi-C data. F1000Res 4, 1310 (2015). 26835000 10.12688/f1000research.7334.1 PMC4706059

[76] Heinz S. Transcription Elongation Can Affect Genome 3D Structure. Cell 174, 1522–1536 e22 (2018). 30146161 10.1016/j.cell.2018.07.047 PMC6130916

[77] Durand N.C. Juicer Provides a One-Click System for Analyzing Loop-Resolution Hi-C Experiments. Cell Syst 3, 95–8 (2016). 27467249 10.1016/j.cels.2016.07.002 PMC5846465

[78] Abdennur N. & Mirny L.A. Cooler: scalable storage for Hi-C data and other genomically labeled arrays. Bioinformatics 36, 311–316 (2020). 31290943 10.1093/bioinformatics/btz540 PMC8205516

[79] Marchal C., Singh N., Corso-Diaz X. & Swaroop A. HiCRes: a computational method to estimate and predict the genomic resolution of Hi-C libraries. Nucleic Acids Res 50, e35 (2022). 34928367 10.1093/nar/gkab1235 PMC8990515

[80] Yang T. HiCRep: assessing the reproducibility of Hi-C data using a stratum-adjusted correlation coefficient. Genome Res 27, 1939–1949 (2017). 28855260 10.1101/gr.220640.117 PMC5668950

[81] Open2C Cooltools: Enabling high-resolution Hi-C analysis in Python. PLoS Comput Biol 20, e1012067 (2024). 38709825 10.1371/journal.pcbi.1012067 PMC11098495

[82] Dixon J.R. Topological domains in mammalian genomes identified by analysis of chromatin interactions. Nature 485, 376–80 (2012). 22495300 10.1038/nature11082 PMC3356448

[83] Roayaei Ardakany A., Gezer H.T., Lonardi S. & Ay F. Mustache: multi-scale detection of chromatin loops from Hi-C and Micro-C maps using scale-space representation. Genome Biol 21, 256 (2020). 32998764 10.1186/s13059-020-02167-0 PMC7528378

[84] Buenrostro J.D., Wu B., Chang H.Y. & Greenleaf W.J. ATAC-seq: A Method for Assaying Chromatin Accessibility Genome-Wide. Curr Protoc Mol Biol 109, 21 29 1–21 29 9 (2015). 10.1002/0471142727.mb2129s109 PMC4374986 25559105

[85] Gaur M. Lactate and histone H3K18 lactylation are associated with metabolic control of gene expression in the retina. PLoS Genet 22, e1012100 (2026). 41950290 10.1371/journal.pgen.1012100 PMC13095125

[86] Langmead B. & Salzberg S.L. Fast gapped-read alignment with Bowtie 2. Nat Methods 9, 357–9 (2012). 22388286 10.1038/nmeth.1923 PMC3322381

[87] Danecek P. Twelve years of SAMtools and BCFtools. Gigascience 10(2021). 10.1093/gigascience/giab008 PMC7931819 33590861

[88] Zhang Y. Model-based analysis of ChIP-Seq (MACS). Genome Biol 9, R137 (2008). 18798982 10.1186/gb-2008-9-9-r137 PMC2592715

[89] Corces M.R. The chromatin accessibility landscape of primary human cancers. Science 362(2018). 10.1126/science.aav1898 PMC6408149 30361341

[90] Ramirez F. deepTools2: a next generation web server for deep-sequencing data analysis. Nucleic Acids Res 44, W160–5 (2016). 27079975 10.1093/nar/gkw257 PMC4987876

[91] Robinson J.T., Thorvaldsdottir H., Turner D. & Mesirov J.P. igv.js: an embeddable JavaScript implementation of the Integrative Genomics Viewer (IGV). Bioinformatics 39(2023). 10.1093/bioinformatics/btac830 PMC9825295 36562559

[92] Hoang T.V., Kizilyaprak C., Spehner D., Humbel B.M. & Schultz P. Automatic segmentation of high pressure frozen and freeze-substituted mouse retina nuclei from FIB-SEM tomograms. J Struct Biol 197, 123–134 (2017). 27725257 10.1016/j.jsb.2016.10.005

[93] Kizilyaprak C., Spehner D., Devys D. & Schultz P. In vivo chromatin organization of mouse rod photoreceptors correlates with histone modifications. PloS one 5, e11039 (2010). 20543957 10.1371/journal.pone.0011039 PMC2882955

